# Immunogenetic and Environmental Factors in Age-Related Macular Disease

**DOI:** 10.3390/ijms25126567

**Published:** 2024-06-14

**Authors:** Sylwia Brodzka, Jędrzej Baszyński, Katarzyna Rektor, Karolina Hołderna-Bona, Emilia Stanek, Natalia Kurhaluk, Halina Tkaczenko, Grażyna Malukiewicz, Alina Woźniak, Piotr Kamiński

**Affiliations:** 1 Division of Ecology and Environmental Protection, Department of Medical Biology and Biochemistry, Faculty of Medicine, Collegium Medicum in Bydgoszcz, Nicolaus Copernicus University in Toruń, M. Skłodowska-Curie St. 9, PL 85-094 Bydgoszcz, Poland; sylwia.brodzka@cm.umk.pl (S.B.); jedrzej.baszynski@cm.umk.pl (J.B.); karolina.holderna@cm.umk.pl (K.H.-B.); emilia.stanek@cm.umk.pl (E.S.); 2 Department of Biotechnology, Institute of Biological Sciences, Faculty of Biological Sciences, University of Zielona Góra, Prof. Z. Szafran St. 1, PL 65-516 Zielona Góra, Poland; k.rektor@wnb.uz.zgora.pl; 3 Institute of Biology, Pomeranian University in Słupsk, Arciszewski St. 22 B, PL 76-200 Słupsk, Poland; natalia.kurhaluk@upsl.edu.pl (N.K.); halina.tkaczenko@upsl.edu.pl (H.T.); 4 Department of Eye Diseases, University Hospital No. 1, Faculty of Medicine, Collegium Medicum in Bydgoszcz, Nicolaus Copernicus University in Toruń, M. Skłodowska-Curie St. 9, PL 85-092 Bydgoszcz, Poland; g.malukiewicz@cm.umk.pl; 5 Department of Medical Biology and Biochemistry, Faculty of Medicine, Collegium Medicum in Bydgoszcz, Nicolaus Copernicus University in Toruń, M. Karłowicz St. 24, PL 85-092 Bydgoszcz, Poland; al1103@cm.umk.pl

**Keywords:** age-related macular degeneration, AMD, chemical elements impact, element-element interactions, oxidative stress, metallothioneins, lifestyle factors, genetic factors

## Abstract

Age-related macular degeneration (AMD) is a chronic disease, which often develops in older people, but this is not the rule. AMD pathogenesis changes include the anatomical and functional complex. As a result of damage, it occurs, in the retina and macula, among other areas. These changes may lead to partial or total loss of vision. This disease can occur in two clinical forms, i.e., dry (progression is slowly and gradually) and exudative (wet, progression is acute and severe), which usually started as dry form. A coexistence of both forms is possible. AMD etiology is not fully understood. Extensive genetic studies have shown that this disease is multifactorial and that genetic determinants, along with environmental and metabolic-functional factors, are important risk factors. This article reviews the impact of heavy metals, macro- and microelements, and genetic factors on the development of AMD. We present the current state of knowledge about the influence of environmental factors and genetic determinants on the progression of AMD in the confrontation with our own research conducted on the Polish population from Kuyavian-Pomeranian and Lubusz Regions. Our research is concentrated on showing how polluted environments of large agglomerations affects the development of AMD. In addition to confirming heavy metal accumulation, the growth of risk of acute phase factors and polymorphism in the genetic material in AMD development, it will also help in the detection of new markers of this disease. This will lead to a better understanding of the etiology of AMD and will help to establish prevention and early treatment.

## 1. Introduction

Age-related macular degeneration (AMD) is a chronic eye disease that usually appears in people after the age of 50, but in highly developed countries it can also occur in younger people. AMD is an important cause of blindness in the USA and accounts for 54% of blindness in Caucasian patients, 4.4% in black patients, and 14.3% in Spanish ones. Among white people aged 40 and older, AMD is the most common cause of visual impairment and blindness in the USA [1]. Due to the increasing life expectancy, it is expected that the frequency of AMD will increase [2,3,4,5,6,7,8]. 

Currently, AMD is considered to be the most prevalent retinal disease in the Western world. Furthermore, it is the most prevalent cause of blindness in developed nations. Considering ethnic factors, the problem is particularly remarkable among Caucasians compared to Hispanic, African, and Asian populations. A large-scale estimation revealed that in 2020, the worldwide population struggling with AMD encompassed approximately 196 million of individuals. The prognosis concerning a longer period of time (by year of 2040) is very alarming, and projects an increase to 288 million individuals. This phenomenon may largely arise from certain diet and lifestyle factors, as well as increasing lifespan [9]. Diverse causes and risk factors of AMD are summarized in Table 1 and Table 2. Neovascular AMD is responsible for 90% of blindness connected with this disease. The mainstream treatment for slowing its progression is anti-vascular endothelial growth factor (anti-VEGF) therapy (aflibercept, ranibizumab). Nevertheless, this method is burdened with disadvantages such as high financial costs, requirement of frequent treatments and finally lack of entire treatment of the disease. Future approaches will be undoubtedly concentrated on the elaboration of new anti-VEGF drugs that conduce to increment in efficacy and prolongation of the treatment intervals, which ultimately make the therapy less expensive [9]. 

Oxidative stress plays an important role in the pathological events occurring in AMD. In this context, there are statements from clinical studies with post-mortem donor eyes that confirm presence of DNA, protein, and lipid biomarkers of oxidative stress in the course of AMD. The occurrence of oxidative DNA damage, which includes 8-hydroxy-2-deoxyguanosine (8-OHdG) is notable, particularly in dry form with RPE atrophy. Similarly, enhanced levels of carboxy-ethyl-pyrrole (CEP) protein adducts (which are indicative of docosahexaenoic acid-containing lipid peroxidation) in the Bruch’s membrane of AMD donor eyes confirms augmented vulnerabilities toward oxidative stress (compared to non-AMD eyes) [12]. 

The dry form of disease may be connected with 60% elevation of CEP levels in comparison with control. A 60% increment in plasma CEP levels may be associated with increased complement factor H, ARMS2 or complement C3. Finally, augmented malondialdehyde (MDA) levels are also reported in serum from AMD individuals, which affirms the systemic elevation of oxidative stress [12]. There are certain observations confirming functionality of compensatory mechanisms of RPE cells to counteract oxidative stress, e.g., antioxidative agents such as catalase (CAT) and superoxide dismutase (SOD) tend to be upregulated in immunoblots of AMD donor eyes, especially in the early or intermediate stages of AMD, demonstrating the existence of a pro-survival mechanism in response to escalated oxidative stress. In cases of wet AMD, there are statements of increment of total oxidant status in serum of patients and decrease of total antioxidant status. Similarly, SOD, glutathione peroxidase (GPx), and glutathione reductase (GR) tend to be decreased in serum of wet AMD individuals compared to healthy individuals, while the activity of SOD, CAT, and GPx also tend to decrease as a consequence of wet AMD when analyzed in the erythrocytes of AMD patients in comparison with control. Therefore, augmented oxidative stress and diminished antioxidative defenses in wet AMD propel further AMD progression and underline the critical role of oxidative stress in the course of disease [12]. 

**Figure 1:**
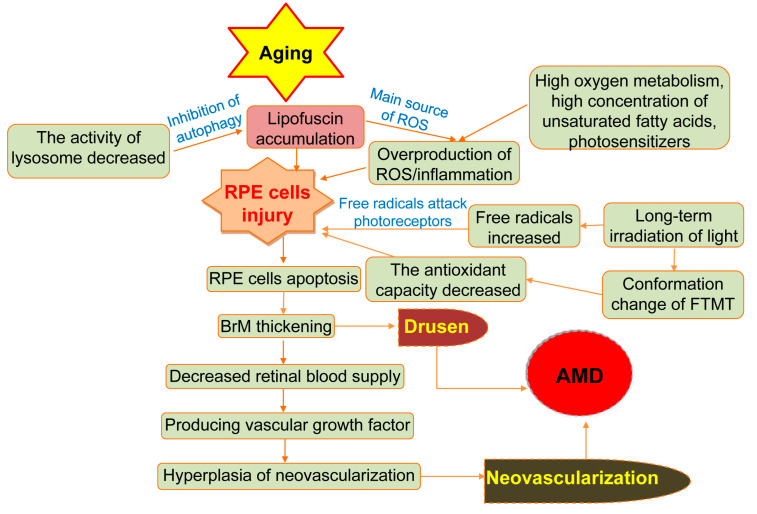
The formation of AMD during aging. Non-genetic mechanisms of AMD are induced by retinal pigment epithelium (RPE) cell senescence, oxidative stress, hemodynamics, and during aging (modified after Deng et al. (2022) [14].

A series of changes caused by aging of RPE cells can lead to AMD. The aging of retinal pigment epithelium cells (RPE) break the balance of enzymes in the extracellular matrix in macular area gathers on the Bruch membrane (BrM). Metabolites accumulate on the BrM, forming vitreous warts, damaging adjacent retinal tissues, and reducing the blood supply of retina. RPE cells senescence leads immune cells to produce vascular endothelial growth factor (VEGF). The calcification, rupture, and phagocytosis of BrM produce blood vessels which may lead to AMD (Figure 1). 

A fundamental factor in the process of neovascularization is VEGF-A, therefore it is also considered to be the the main focus of anti-VEGF therapies. Pathological angiogenesis is connected with the improper regulation of VEGF, while this factor also promotes proliferation and tube formation, as well as the migration and vascular permeability of endothelial cells. The fact that RPE releases VEGF in the fetal development is necessary in the development of choriocapillaris. Furthermore, low ocular level of VEGF generally is considered natural under physiological conditions [13]. In turn, pathological conditions may be characterized by abnormally high levels of VEGF in affected zones, which may indicate advances in neovascularization. Another characteristic feature for AMD patients is improper regulation of para-inflammatory responses connected with the promotion of macular damages. These processes are additionally stimulated by certain behavioral risk factors, genetic predispositions, and advanced age [13]. 

**Figure 2:**
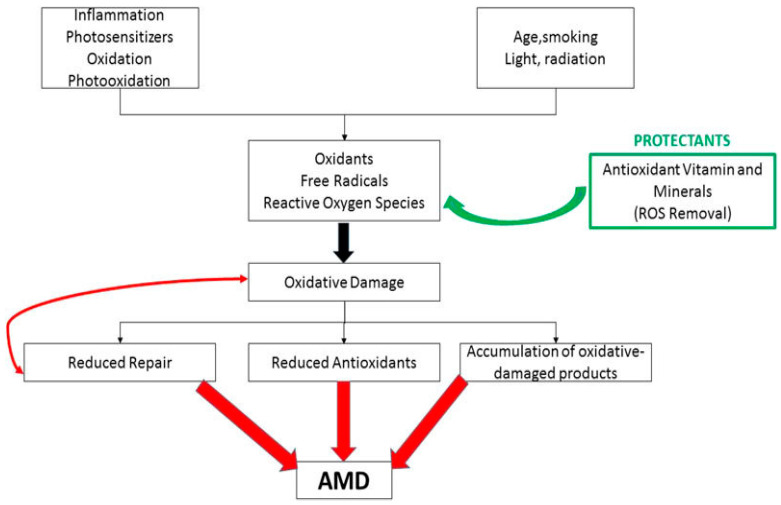
AMD development and protective mechanism of antioxidants (modified after Arslan et al., 2018 [17]. Arrows indicate mutual relations between parameters in rectangles.

The precise mechanism of this impact is still unknown. Previous studies have shown that, due to pathological changes in retinal pigment epithelium (RPE), the macula is damaged, which may lead to partial or complete loss of vision. The disease occurs in two clinical forms, dry and exudative (wet, neovascular), while the dry form tends to convert into a wet form. A dry form of AMD is a mild and gradual disease. However, it displays an acute and violent nature in the neovascular form. Both forms of AMD can occur simultaneously [3,4,5,6,7,8]. Environmental and genetic factors contribute significantly to the development of AMD. Studies have confirmed the role of heavy metal ions, chemicals, light, and many genes that increase the risk of disease [5,7,8] (Figure 2). 

In context of immune surveillance, there is also a critical role of complement system because its overactivation may lead to AMD. Therefore, certain complement inhibitors are in advanced clinical trials for the treatment of AMD. Such therapies can be applied directly to the eye by intravitreal, subretinal, and suprachoroidal injections, as well as delivered systemically. However, high-resolution ophthalmic imaging revealed that the study of BrM appears fundamental for understanding of AMD pathogenesis, because BrM is responsible for key transport functions and constitutes structural support to RPE, while, in the process of aging, it undergoes detrimental structural changes including thickening, decreased elasticity, and reduced permeability. Thus, perception of transit of complement proteins and vectors across BrM and RPE is also pivotal for the efficacy of therapies based on application of complement inhibitors and regulators [15]. In cases of therapeutic use and possible clinical applications, certain biomaterials are praised for their potential in drug release control, redistribution, and the establishment of long-term delivery. In AMD therapy, certain port delivery systems (PDSs) have achieved long-term performance in delivering VEGF-A antibodies. 

Additionally, the application of specifically fabricated micro-boxes may facilitate long-term delivery of various types of therapeutics. Finally, nanobiomaterials are able to control the intracellular inflammatory signals by acting as ROS scavengers and redox balance stabilizers. Such nanoplatforms may deliver anti-inflammatory agents to the disease regions, e.g., anti-VEGF-A antibody-loaded betasone phosphate-based hydrogel guarantees not only prolonged attenuation of choroidal neovascularization but also scavenges ROS to reduce inflammations [16]. 

Previous studies indicate that the impact of heavy metals leads to accumulation of reactive oxygen species (ROS). Unfortunately, there is no clear evidence that oxidative stress is a major factor in AMD development, but ROS continues to be an important factor in the course of disease [3,4,5,6,7,8,18]. ROS is a group of compounds of hydrogen peroxide (H_2_O_2_), singlet oxygen (^1^O_2_), superoxide radical anion (O_2_*^−^), and hydroxyl radicals (*OH) [3,4,5,6,7,8,19]—and the harmful activity of ROS damages lipid membranes, proteins, and nucleic acids [3,4,5,6,7,8,20,21,22]. The most exposed element for oxidative stress is the retina. This is due to its continuous exposure to radiation, high concentrations of O_2_, high levels of polyunsaturated fatty acids in external photoreceptors, and the presence of many chromophores (lipofuscin, melanin, rhodopine, cytochrome C oxidase) in the retina and retinal pigment epithelial cells RPE generating ROS during phagocytosis of photoreceptor disks by RPE [3,4,5,6,7,8,19,20,22]. Molecules damaged by ROS called advanced glycation products AGE affect cell DNA and increase expression of genes that promote aging of RPE cells [23].

To counteract the effects of oxidative stress, a response is triggered in two stages. The first one is the reaction where cytochrome P450 monooxygenase system is involved. The action involves the oxidation and reduction of dangerous compounds. In the next phase, these products are coupled with hydrophilic molecules. Antioxidants involved in the second phase are divided into “direct” and “indirect”. The “direct” group includes SOD, glutathione GSH, and thioredoxin TR. The first two components oxidize dangerous compounds and quickly regenerate themselves. The “indirect” enzymes participate in the biosynthesis and regeneration of GSH and TR. They also participate in the removal of oxidized compounds [24]. The nuclear factor erythroid-2 related factor 2 NrF2 plays a major role in the antioxidant response. It activates transcription of the leucine slider by binding to the antioxidant response element ARE within the promoter of target genes. Its role is to maintain redox homeostasis in the cell [4,6,24,25,26,27]. NrF2 regulates both so-called “direct” and “indirect” enzymes [24,28]. The main task of this factor is to induce antioxidant response [4,24,27]. NrF2 precisely controls the activation of antioxidant genes in response to oxidative stress, which affects cell survival. Any defects in the antioxidant defense, controlled by NrF2, can contribute to the cell death by apoptosis. NrF2 signaling play an important role in the functioning of RPE cells [4,24,29,30,31].

Retinal cells are exposed to oxidative stress due to a large extent to light effects [3,4,5,6,7,8,19,20,22]. As a result of the increasing aging processes, cells do not respond to mitogenic factors and lose their ability to proliferate. Studies have confirmed the presence of AGE-exposed genes in RPE cells, as well as the occurrence of shorter telomeres [32]. AGE undergoes metabolism in a much more difficult manner, and as a result they are deposited in the lysosomes in the form of lipofuscin, which, in turn, is deposited in the form of drusen [33]. As a result, this disrupts the metabolic exchange between the choroid and retina [34]. Additionally, the work of photoreceptors is weakened [35]. Then, a low intensity inflammatory process begins, which increases the levels of acute-phase proteins, reducing total antioxidant status (TAS) [3,4,5,6,7,8,20,22,36,37,38,39]. The next stage is neovascularization, which occurs due to the secretion of proangiogenic factors during para-inflammatory process. New capillaries come from the choroid and are formed under the retina in the area of macula, where they should not occur [7]. As a result, photoreceptors are destroyed in the macula [3,4,5,6,7,8,20,22]. 

Thus, taking into account the above interrelationships, we consider the following aspects:

1. Presentation of the current state of knowledge about the impact of environmental and genetic factors on the development of AMD in relation to our research conducted on the Polish population in the Kuyavian–Pomeranian and Lubusz Regions (Central and SW Poland).
2. Analyzing how the polluted environment of large agglomerations affects the development of AMD, because this disease has only recently been a research subject and its etiology is still not well known.
3. Indication of new markers, because the review of the current literature from this field is very optimistic, which means that emphasis are changes in the protein and enzyme activity, metal concentration, gene polymorphism. This is the starting point for the analyses that we intend to perform within our studies. We investigated concentration of chemical elements Na, K, Ca, Mg, Be, V, Cr, Mn, Fe, Co, Ni, Cu, Zn, Al, Si, P, S, As, Se, Mo, Cd, activity of antioxidant enzymes (SOD, CAT, GPx, GR); intensity of lipoperoxidation (MDA), stress proteins (ceruloplasmin CP, haptoglobin HPT, ferritin FRT), non-enzymatic mechanisms (total antioxidant status TAS, uric acid, bilirubin).
4. Analysis of all the above relationships, which can bring us closer to a multi-level response to the question of the causes and development of pathophysiological changes that accompany AMD.

## 2. Role of Toxic Heavy Metals in AMD Development

As heavy metals are characterized by greater atomic mass than sodium atomic mass, with a density greater than 5 g·cm^−3^ and include elements of semi-metals (metaloids, e.g., As, Sb), they are classified as most important environmental and occupational poisons. As, Cr, Cd, Ni, Pb, Hg, Cr, Ni are highly allergenic [40]. High concentrations of heavy metals induce immunosuppressive effects on the immune system, while low concentrations cause immunostimulation. Metal ions cause oxidative stress in different ways, e.g., Fe, Cr, and Cu undergo redox reactions by increasing ROS leading to their over-production. In turn, Cd, Pb, and Ni decrease the antioxidative potential of cells, leading to ROS accumulation due to their reduced neutralization [41].

Metal ions have less molecules than organic chemicals, and the bonding they create is not a permanent covalent bond but a reversible one. In this case, the activation of immune cells by metal ions may take place in a manner different from that of classical hapten. Metal ions with high reduction and oxidation potential, such as Au (IV), can lead to irreversible denaturation of proteins by oxidation of sulfur-containing side chains in amino acids [42]. Metals primarily induce changes in protein synthesis, damage cell membranes and impair ATP production. Most react with sulfhydryl, carboxyl, and phosphate groups. The processes taking place in the cell depend on the amount of metals and their degree of affinity. The changes in metabolic processes in the body are revealed in the form of biochemical or clinical effects. Their occurrence is related to the critical concentration of elements in the organs [43]. 

Much research has been devoted to the impact of heavy metals on the nuclear factor erythroid-2 related with factor 2 (NrF2). Mice experiments have shown that, in response to light toxicity in vivo, thioredoxin is induced via thiophene [24,44]. In the experiments conducted by Gao and Talalay (2004) [45], human ARPE-19 cells were used, the sulforaphane was isolated by HPLC and concentration measured spectrophotometrically. The results indicated activation of NrF2 sulforaphane as a protective factor for RPE. One year later, Western Blot and immunohistochemically tested on human RPE K-1034 cells were assayed using murine cells and analyzed by flow cytometry and electrophoresis. The results confirmed activation via NrF2 and showed direct protection of RPE by the alpha-tocopherol and intermediate by inducing thioredoxin [24,44]. Glutathione synthesis is also induced by this factor by the activation of glutathione S-transferase and NADPH-quinone reductase.

Research conducted by Nelson et al. (2002) [46] included hRPE cells cultured at different concentrations of oltipraz and were subjected to a chemical oxidant. The level of glutathione in the mitochondria was determined by the HPLC method, and glutathione reductase activity was determined spectrophotometrically. The result was a significant increase in GST activity caused by the compound used. Research has speculated that oliprase may bind to glutathione and then to the nucleophilic center of transcriptional regulatory factor, and by interacting with NrF2, affecting the transcription of enzymes in the second phase of antioxidative response [24]. Conversely, studies by Ha et al. (2006) [47] tested the protective effect of zinc on RPE. The experiment was carried out on human ARPE-19 cell culture. Isolation of glutathione was made by HPLC. Gene expression was tested using RT-PCR. As a result, zinc has been shown to increase the amount of glutathione in RPE cells, and through NrF2 increases its de novo synthesis and protects RPE [24]. The impact of toxic metals on humans is presented in Table 3.

### 2.1. Element-Element Interactions in AMD

According to research focused on the impact of lead, it was positively associated with both early and late AMD form in all studied local and regional areas. Hg and Cd also had a positive relationship with late AMD in all studied areas, but did not affect early AMD. In contrast, Mn and Zn were the opposite of late AMD [58]. Mn and Zn are not associated with early AMD [59]. Pb and Cd can accumulate in human retinal tissues which may also be damaged in result of oxidative stress. They may therefore play a role in the development of AMD. AMD has a great significance as a cause of blindness in the USA (that accounts even for 54% of blindness in Caucasian patients, and is also considered an important risk factor for black patients, and people from Spain). Certainly, age and ethnic determinations play important role and among white people aged 40 and older, AMD is the predominant reason of visual impairment and loss of vision in the US [1]. Due to increasing life expectancy, the incidence of AMD is expected to increase [3,4,5,6,7,8].

Studies have shown that lead and cadmium can promote chronic disease, increasing chronic oxidative stress by damaging DNA, reducing antioxidant capacity of defense systems, stimulating the production of inflammatory cytokines [60], and ROS production, also in retinal pigmented epithelial cells (RPE) [3,4,5,6,7,8,21,57]. Cells involved in the transport of trace metals such as Pb and Cd are particularly susceptible to toxicity. RPE can interact with metals, including basic metals such as Zn, Cu, Fe, but also with toxic heavy metals, including Pb and Cd, which have a high affinity for melanin in RPE melanosomes [61,62]. Pb and Cd ions are similar in size to Zn, Cu, and Fe. Cd concentrations in human eyes increase with age and are higher in women than in men and in smokers compared to non-smokers [56,57]. Pb concentration in human retina also increases with age [56]. 

Problematic matter is detection of presence of multiple toxic metals within the intact retina. It is also difficult to separate primary metal toxicity from secondary uptake of metals in defective tissue. However, elemental bioimaging enables detection of toxic metals in various segments of eye [63]. Certain examinations use such methods as laser ablation-inductively coupled plasma mass spectrometry that make possible the detection of multiple elements in tissues as well as histochemical technique of auto-metallography that allow to detect the presence of inorganic Hg. Therefore, the presence of such toxic elements as Ni, Fe, Hg, and Al can be confirmed in retinal pigment epithelium, choriocapillaris or optic nerve head. Subsequently, this knowledge is fundamental for inferences about injuries to RPE from toxic metals, damages of neuroprotective functions or violations of outer neural retina [63]. Due to the fact that the degree of bioaccumulation of heavy metals in human body is high, there is a constant need to monitor their levels and influence. The development of biological methods of identification and quantification as well as usage of biomarkers create a possibility of establishment of reliable criteria and standards. It gains importance because the retinal epithelium is a chelating metal tissue, while melanin forms bonds with heavy metals that increase tissue affinity for these elements. Finally, wide variety of anthropogenic activities have led to the continuous release of toxic pollutants into the environment and made this problem so urgent [64]. 

### 2.2. Accumulation of Heavy Metal Ions in Eye Tissues 

Research on human eyes confirmed the ability of the retina and choroid to accumulate heavy metals [65]. Analyses carried out on human eyes using X-ray showed that sometimes Al, Hg, and Se may be present in melanosomes in retinal pigment epithelium. In turn, lead spectra were observed in the choroid and retina. Accurate studies to determine the limit values of heavy metals for humans were carried out in 2005. The ability of Pb, Cd, Tl, and Hg metals to accumulate in the tissues of human eye has been confirmed. Studies were conducted using plasma-mass spectrometer and histologic examination. It has been shown the greatest affinity of these metals to the pigmental epithelium of retina and choroid. Pigmentary eye tissues, such as retina pigmentary epithelium, choroidal, iris, and ciliary body, have a high affinity for heavy metal ions. Additionally, melanin present in pigmented granules binds metal ions that bind to melanosomes according to atomic weight and volume [56,61]. It has also been proven that heavy metals, such as Pb and Cd, interfere with biochemical balance in cell and cause oxidative stress resulting from the production of ROS [21,56,66].

The occurrence of AMD correlated with cutaneous signs of Hg toxicity with skin lesions induced by toxic influence of this element [67]. Both histopathological studies of the skin biopsy, fundus, and determination of Hg concentration in the blood were performed. Experiments were performed on the Grover’s disease patients stated that skin lesions in the course of the Grover’s disease may be an AMD marker. In addition, even low levels of mercury correlate with development of both diseases. However, the author also pointed out that if the results are confirmed in other studies, the elimination of Hg from the environment reduce the number of patients on both units [67].

Studies conducted in 2005 on the Caucasian race have demonstrated the presence of Pb and Cd in all examined eye tissues. Their concentration was higher in the retinal pigment epithelium/choroid than in the retina. The work involved histological examination and measurement of metal concentrations in tissues and blood by mass spectroscopy. As a result, it has been demonstrated that in the retinal pigment of retina and choroid, as well as in ciliary Cd and Pb of retina occur at the concentrations higher than in the blood and the fluid of the eye. The results confirmed the ability of Cd and Pb to accumulate in RPE and choroid. However, there are no known diseases that can be caused by this phenomenon [56].

Very important amphoteric chemical element, aluminum, was tested to correlate with AMD development. Studies have shown that Al salts activate the NALP3 inflammasome, causing lesions and rupture of lysosomes as well as migration of immune cells and inflammation. As a result, tissue damage and tumor growth occurred. Studies were conducted on mice using flow cytometry, confocal microscopy, as well as immunochemical methods and Western Blot [68]. Additionally, Cd and Pb concentrations in human eye tissue were studied. As a result, it has been shown that Pb accumulation is related to AMD’s development and significantly affects homeostasis of the eye tissue. The neural cells of retina with AMD had increased Pb concentration. In turn, Cd concentrations in patients were not significantly higher than in healthy patients [69].

In 2008–2011, Korean team measured concentrations of Hg, Cd, Mn, Zn, and Pb in human peripheral blood in correlation with AMD. The results showed that high concentrations of lead were associated with development of both early and late AMD. In turn, Hg and Cd only correlated with pathogenesis of late AMD. The results for Mn and Zn were different; their high concentrations had protective effects in the late form of AMD [59]. Other studies have identified Zn as another potential protective factor for AMD, even in patients with polymorphisms that increase the risk of AMD development [5,70].

## 3. Role of Oxidative Stress 

Cellular imbalance occurs as a result of oxidative stress. This is caused by an elevated oxidation reaction that produces ROS (superoxide radical anion (O_2_*^−^), hydroxyl radical (*OH), hydrogen peroxide (H_2_O_2_), and singlet oxygen (^1^O_2_). ROS can be formed in many ways as a product of respiratory chain in mitochondria, in photochemical or enzymatic reactions, as effect of exposure to UV light, ionizing radiation, or the influence of metal ions. Hydrogen peroxide is a low reactivity molecule, but it can easily penetrate cell membranes and generate the most reactive forms of oxygen, i.e., hydroxyl radical through Fenton’s reaction [3,4,5,6,7,8,20,21,22]. 

ROS play an important role in the regulation of many physiological processes by participating in intracellular signaling [21,71], and causes serious damage of biomolecules by lipid peroxidation. This is a process of oxidation of polyunsaturated fatty acids due to the presence of several double bonds in the structure and involves the production of organic peroxides and reactive free radicals. These molecules react with other fatty acids, and initiate a chain reaction. ROS also attack structural and enzymatic proteins by oxidation of amino acid residues, a formation of transverse bonds and protein aggregates as well as proteolysis. Deactivation of key proteins can have serious consequences in important metabolic pathways. ROS can also react with nucleic acids (attacking nitrogenous bases and phosphate skeleton of sugar), and can cause damage to single- and double-stranded DNA. The inability of the cell to repair damage may lead to death, or alternatively, there may be mutations in the DNA, which leads to the development of cancer or neurodegenerative diseases [3,4,5,6,7,8,20,21,22,72].

Oxidative stress, causing damage to the eye, can occur in various forms and can be stimulated by various factors [3,4,5,6,7,8,20,21,22,73]. In human eyes, local exposure to light combined with locally high oxygen content, which is greater than in other tissues, leads to a high tendency for oxidative stress. In combination with systemic exposure to oxidative stressors, conditioned by lifestyle or other factors, relative oxidative stress can quickly become disproportionately high. Oxidative stress is associated with the promotion of inflammation and has a number of negative effects associated with AMD progression [3,4,5,6,7,8,20,21,22,54,73,74,75,76,77]. As a result of the recognition of system disorders, anomalies in DNA repair genes, inefficient repair of DNA damage, and acceleration of aging of the organism occur, leading to the dysfunction of cells and tissues. The aging is unavoidable because ROS formation is the result of normal, daily cellular metabolism. In effect, the cells have developed the complex of defense mechanisms to fight both the resulting of ROS and their effects [3,4,5,6,7,8,20,21,22,54]. Cells most susceptible to oxidative stress damage are non-proliferating postmitotic cells, including photoreceptors and RPE cells, because they do not have any DNA damage detection systems at the cell cycle checkpoints [3,4,5,6,7,8,20,22]. 

RPE is a retinal epithelium, a pigmented monolayer, located between the retina and the choroid. RPE is essential for the preservation and survival of photoreceptor cells because it performs a number of critical functions such as the formation of an external retinal barrier, transport, retinoid retention, phagocytosis, degradation of segmental photoreceptors, and protection against light and oxidative stress [78]. Contaminated environment promotes ROS production [3,4,5,6,7,8,20,22]. In the macula, the dominant photoreceptors are cones, which exhibit higher demand and energy production than rods, and therefore higher oxygen requirements [79,80,81,82]. Rod cells and cones differ in their susceptibility to oxidative stress, and cones show greater sensitivity to free radicals [83]. Macula is consistently exposed to a high metabolic activity and oxidative stress due to a high partial pressure of choriocapillaries and polyunsaturated fatty acids (PUFAs) from external segments of the retina [3,4,5,6,7,8,20,22,54,84]. This is considered in inducing drusen formation between RPE cells and Bruch’s membrane. 

Lipofuscin is a chromophore serving as the primary RPE photooxidation agent [85] which, after absorbing high energy photons, especially blue light, undergoes a series of photochemical reactions involving ROS formation, which in turn induces photochemical damage in the retina and RPE cells [3,4,5,6,7,8,20,22,54,86]. Autophagocytosis and RPE homeostasis pathways play an important role in the aging of cells and affect oxidative stress in AMD. Autophagy, cellular and molecular pathways were investigated for oxidative stress. The results indicated that acute exposure to oxidative stress increased autophagy activity, while chronic exposure to oxidative stress reduced autophagy. Based on murine and human models, it has been observed that autophagy is significantly elevated in early AMD, while its activity decreases in late AMD. Moreover, it has been observed that reduced autophagocytosis effect makes RPE more susceptible to oxidative stress and that autophagy system is enhanced to protect RPE from oxidative damage [78]. 

Oxidative stress in retina may be triggered by chronic UVA exposure, while blue light exposure also promotes this phenomenon in retinal pigment epithelium layer. In the unfavorable conditions results may be deepened by cigarette smoke or aging process. Another factor contributing to oxidative stress in RPE is phagocytosis of outer segments of the retina, which are abundant in polyunsaturated fatty acids. Subsequently their oxidative alteration cause formation of carboxy-ethyl-pyrrole adducts as well as advanced glycation end-products which may be detected within drusen. Ultimately, the accumulation of lipofuscin occurs within the lysosomal compartments and lysosomal degradation undergoes disturbance that results in amassment of protein and lipid extracellular deposits. Existence of certain non-invasive imaging techniques like optical coherence tomography (OCT), fundus autofluorescence or OCT angiography enable the insight into key retinal and choroidal anomalies. This makes possible the characterization of AMD features (drusen, neovascularization, GA) and investigation of disease advancements [87].

## 4. Role of Metallothioneins in AMD

Metallothioneins are low-molecular-weight (6–7 kDa) intracellular proteins, which were firstly isolated from horse kidneys and appreciated for high potential in binding cadmium [54,88,89]. These proteins are able to bind metals and act as free radical scavengers. This ability result in considering metallothioneins as one of important components of antioxidative defense (beside typical enzymatic mechanisms like dismutases or catalases, as well as nonenzymatic like glutathione, ascorbate, or carotenoids) [20,21,22,88]. These proteins have two domains, α and β. The α-domain (consists of 11 cysteine residues) is able to bind four Zn^2+^, four Cd^2+^, or six Cu^+^ ions. The β-domain (consists of nine cysteine residues) can binds three Cd^2+^, three Zn^2+^, or six Cu^+^ ions [21,22,88]. Metallothioneins are also stabilized by interactions with other metal ions such as Hg, Pb, Ni, or Co [54]. Single molecule of metallothioneine may contain 12 Cu atoms, as well as 7 Cd or Zn atoms [21]. 

Metallothioneins were identified in many cellular structures (nucleus, cytosol, lysosomes, mitochondrial intermembrane space). A total of four isoforms of metallothioneins (MT-1, MT-2, MT-3, MT-4) have been identified in the human body. Their location is presented in Table 4. The first two (MT-1 and MT-2) undergo expression in many tissues, while the expression of the third one (MT-3) is concentrated on central nervous system. The expression of all three isoforms was detected in the human brain and retina [20,21,22,54,88]. The construction of metallothioneine molecules (content of metals and thiol groups) enables them to make quick interactions with ROS (hydroxyl radical and superoxide anion radical) [21]. 

Through capturing of cations (like Fe) metallothioneins prevent from completing Fenton reaction, in which ROS may be generated [20]. In this reaction, hydrogen peroxide reacts with iron (II) ion creating hydroxyl radical (one of the most reactive oxidants). The high redox potential of this molecule enables it to interact with practically all substances present in the organism [21]. The ability of metallothioneins to counteract against oxidative stress as well as their presence in retina prompt to reflection about the role of these proteins in the process of AMD development, strictly connected with age-related progressive degenerative changes in retina [3,4,6,7,8]. A certain chance of counteraction against these destructive changes appeared to be possible with the introduction of antioxidants (vitamin C, E, β-carotene) to the patient’s diet or zinc supplementation [4,5,88]. Integrated action of antioxidants and Zn is able to slow down AMD progression and metallothioneins by binding Zn participate in this process as well [5,88]. Metallothioneins are able to interact with retinol dehydrogenase through this participation in retinol reconstruction in the visual cycle [6]. Alvarez et al. (2012) [22] describe the close correlation between zinc and metallothioneins expression. According to these relations, the presence of Zn increases the expression of metallothioneine, simultaneously decreasing expression of inflammatory cytokines. 

It was noted that two isoforms of metallothionein (MT-1, MT-2) come under particularly high expression in the front structures of eye in range of cornea and lens. High expression of metallothioneins in this area creates natural antioxidative barrier in the structures subject for light, UV, or other kind of radiation [88]. MT-3 is expressed at high levels in the neurosensory retina. This isoform is involved in CNV and vascular leakage from CNV. Moreover, MT-3 is able to protect the outer nuclear layer and photoreceptor cells related to dry AMD [89]. However, increasing the metallothineins expression cause enlargement in amount of bounded Zn [22]. In this mutually regulated mechanism, we can see the potential of metallothioneins to protect the retina against oxidative stress, particularly in the retinal pigment epithelium, neurons, and photoreceptors. The increment of metallothioneins secretion in these regions is a typical defense reaction against growing oxidative stress [20,22,88]. This way, the risk of extension of inflammation to internal structures of eye is reduced. Therefore, two roles of metallothioneins (antioxidative and anti-inflammatory) are replenished [22]. An additional trump of metallothioneins is the considerable speed of interaction with superoxide anion radical or hydroxyl radical. Thanks to this elimination of oxidative stress, precursors may occur effectively [21]. 

Swindell (2011) [54] consider metalthioneins to be “universal free radical scavengers” that take part in cellular response for diversified stress conditions. In his opinion antioxidative potential of metallothioneins confirm their significant role, especially in diseases which grow more intense in advanced age. Oxidative stress itself may be considered to be a group of factors accelerating biological aging of cells. Swindell (2011) [54] also enumerates some studies confirming a regulatory influence of metallothioneins on cellular respiration. Metallothioneins act on two levels, participating in the detoxification of free radicals, as well as not permitting to their rise [54]. There is evidence (via animal models) confirming that these proteins may positively influence the length of life, as well as that of the expression of metallothionein increases in response to calorific restriction (undernutrition), and in unfavorable or toxic conditions [54].

In addition to regulatory function for toxic metals, metallothioneins counteract against cellular apoptosis, alleviate cellular inflammatory processes, support growth, and sedate cell metabolism [54]. All mentioned functions of metallothioneins apply to protection of nervous system cells as well. This way, metallothioneins support the regenerative processes occurring in the nervous system in response to widely undrstood damages [20,22,54]. These statements may be referred to as AMD (as a degenerative disease, which after-effects intensify in advanced age, leading even to complete loss of vision sense) because metallothioneins appear to support the survival of cells and the structures of organism through a counteraction against the aging processes stimulated by oxidative stress [3,4,5,6,7,8]. Thus, metallothioneins eliminate one of the important factors of oxidative stress stimulation by binding and the detoxification of heavy metals. The binding of Zn and Cu favors the maintenance of homeostasis of these elements. There is some mutually regulated mechanism between zinc and metallothioneins; they bind Zn, and moreover, exposure to Zn promotes the production of metallothioneins. The increase in the synthesis of metallothioneins (MT-1, MT-2, MT-3) also occurs in inflammatory conditions in response to the increased level of inflammatory cytokines [20,21,22,54,88,89].

## 5. Lifestyle and Environmental Risk Factors

### 5.1. Smoking 

Different studies show that smoking is one of the most common causes of change leading to AMD. There are thousands of chemicals in the cigarettes, which have harmful influences on the organism. Numerous substances increase the level of oxidative stress, which lead to lipid peroxidation [10,90,91,92,93,94]. According to Velilla et al. (2013) [90], smoking affects the occurrence of molecular and pathological changes, which very strongly affect the macula and decide on the incidence of AMD. Studies showed that patients with AMD have a higher concentration of C-reactive protein (CRP). Unfortunately, a higher level of CRP is not specific to smoking; concentration of this protein increases during inflammation in the body. Research by Seddon et al. (2004) [95] and Molins et al. (2018) [96] showed that a higher level of CRP can increase the risk of AMD independently of smoking. 

Smoking has multiple influence on the development of AMD. Nicotine from cigarettes stimulate the organism to produce vascular endothelial growth factor (VEGF). This factor has proangiogenic activity, which promotes angiogenesis. This process has an important role in the development of wet AMD. Smoking causes inflammation by decreasing concentration of complement factor H (CFH) in the blood and activating inflammatory mediators (e.g., complement C3) [90,91,92,93,94,95]. Environmental independent studies from Australia, Europe, and North America prove the role of smoking in the development of AMD [90]. Research by Thornton et al. (2005) [91] suggests a causal relationship between cigarette smoking and various types of AMD obtained by epidemiological evidence. The results show that the risk of AMD among smokers is three times greater in comparison to non-smokers. This difference is even greater in cases of exudative AMD, and is up to a four-fold increase in the risk of disease [91]. However, most patients and individuals do not show that they increase their probability of the development of AMD by smoking cigarettes, which may lead to total vision loss. Fortunately, for many smokers, the risk of blindness is the most mobilizing factor to quit smoking [91]. It has been confirmed that smoking cessation reduces the risk of developing AMD. Research proves that 20 years after the end of smoking, the risk from cigarette substances decreases to zero, as in people who had never smoked [10,90,97].

### 5.2. The Impact of Light

The light is form of electromagnetic energy, part of this spectrum, which interacts with the eye is referred to as optical radiation. This spectrum includes wavelengths from ultraviolet (UV, 100–400 nm), visible light (400–760 nm), and infrared (IR, 760–10,000 nm). In this optical radiation the Commission Internationale de l’Eclairage had defined several subgroups of wavelengths with similar photon energy. Today, UV radiation is classified into three subgroups: UVA (315–400 nm), UVB (260–315 nm), UVC (100–260 nm), and UVD (20–100 nm). Infrared light (IR) consists of three groups: IRA (700–1400 nm), IRB (1400–3000 nm), and IRC (3000–10,000 nm) [98,99]. 

The spectrum of light that reaches the eye is absorbed by its various tissues. The cornea absorbs almost all radiation below 295 nm. The lens absorbs wavelength range from 300 nm to 400 nm, which is part of UVB (315–400 nm) and total UVA [99,100,101]. The vitreous gel propagates wavelengths in the visual spectrum (400–700 nm) and IRA (700–1400 nm), but the UV, IRB, and IRC bands are almost entirely absorbed. The remainder of the optical radiation, which has a range of 400 nm (380 nm) to 1400 nm, reaches the retina of the eye and is referred to as the retinal hazard region [99,101]. The light can damage the retina by the photothermal, photomechanical, and photochemical pathways [99]. In cases of AMD development, the most important is the last one, in which oxidative stress plays a key role [74,102]. The retina’s oxygen-rich and photosensitizer-rich environment combined with constant radiation exposure is perfect for the production of extremely lethal ROS [103]. 

Susceptibility to damage depends on the wavelength, which is inversely proportional to the energy it delivers. Studies show that the most important is high frequency and high energy visible light of 400–500 nm blue-violet band, referred to as blue light or high energy visible light (HEV). The ability to cause retinal damage as a result of HEV light is known as the blue light hazard (BLH) [100,101]. Green light, having a longer wavelength (495–570 nm) and lower energy, is 50–80 times less phototoxic than blue [104]. In studies by Braunstein and Sparrow (2005) [105], as well as Remé et al. (2000) [106], in vitro cell cultures, under the influence of green light, in contrast to those illuminated with blue light, were not damaged [105,106]. Having an even lower frequency, infrared radiation needs as much as 1000 times more power to produce similar phototoxic effects like HEV light in a given unit of time [107]. 

Epidemiological studies to assess exposure and associated risk of AMD are difficult to construct. The problem is a thorough estimation of exposure, as well as the potential impact of many blackout factors [108,109]. Nevertheless, Taylor et al. (1990) [110] studying of over 800 people working at the Chesapeake Bay found a significant correlation between exposure to blue light or the full range of visible light, and macular degeneration [110]. The Beaver Dam Eye Study published in 2004 also found a significant correlation between increased sunlight accumulation and a 10-year risk of early onset of macular degeneration [111]. European Eye Study (EUREYE), carried out for nearly 5000 participants aged 65 years or older demonstrated a positive correlation between AMD and exposure to blue light in people with low levels of antioxidants [102]. 

Conversely, in a large project of Pathologies Oculaires Lieesa l’Age (POLA), in Sète (S France), unlike the earlier mentioned, there was no significant relationship between exposure to sunlight and the development of AMD [112]. A meta-analysis published in 2013 showed that individuals with higher levels of sunlight exposure have significantly increased risk of AMD. In the same analysis, the latitude could be a covariate, which is negatively correlated with the strength of association, but the meta-regression had no sufficient evidence to support the thesis [108]. 

### 5.3. Diet and BMI

Type of diet appears important, e.g., the adherence to Mediterranean diet generally reduces the risk of developing late AMD (this diet characterizes itself with high content of antioxidants and very limited consumption of red meat). Conversely, consumption of a high-glycemic-index diet may pose a risk factor for the development of AMD [10]. AN unbalanced western diet may deliver additional dietary oxidants creating the risk of elevated ROS in RPE. Conversely, there are some statements that the association of early AMD prevalence tend to decrease with plant and sea-food-based diet. High-fat consumption certainly creates a risk of progression of AMD. Such a threat may be connected both with animal-based or plant-based high-fat diet and is associated with intensification of oxidative stress [113]. It has been confirmed that the role of nutritional factors in modulation of risk of developing AMD is significant. 

Certain expectations are connected with administration of antioxidants because there were some promising effects of nutritional supplementation with vitamins C, E, β-carotene, Zn in delaying of AMD progression and alleviating oxidative stress. The usage of lutein and zeaxanthin replacing β-carotene appeared beneficial in context of intermediate-stage AMD. However, the effectiveness of such approaches is still controversial. Especially there are discussions about genetics–nutrient interactions and the usage of supplements in different ethnic groups [113]. The products containing β-carotene and omega-3 acids are known for their protective role against the development of AMD [10,114,115,116,117], because of their antioxidant, anti-inflammatory and anti-angiogenic effects [114]. It is also known that taking dietary supplements containing lutein, zeaxanthin, antioxidants (vitamin A, C, E and others) or minerals factors protect against the development of wet AMD [10,74,114,117,118]. Therefore, a diet low in antioxidants is one of the risk factors for AMD development [10,119]. However, supplementation of antioxidants is justified only in people, which are suffering from advanced form of AMD. 

Unfortunately, people suffering from the early form of AMD will not reduce the chances of this disease by increasing intake of antioxidants. In turn, lipids (cholesterol, triglycerides) may accumulate in Bruch’s membrane, which may result in the formation of drusen. Therefore, a diet rich in fats increase the risk of developing AMD [10,119]. Additionally, eating high-glycemic-index products may be a risk factor for the development of AMD [10]. 

Studies showed that a higher BMI correlates with the development of AMD [10,107,114,119]. Individuals with BMI higher than 30 have an especially higher risk of developing late AMD than individuals with BMI in normal range (20–25). Obese individuals also tend to demonstrate elevated level of certain pro-inflammatory factors, as well as downgraded antioxidative possibilities that perturbs the functionality of RPE and escalate the development of AMD [10]. Therefore, overweight has a significant contribution in the increasing of risk development of AMD, because obesity is a pro-inflammatory state, promoting the development of oxidative stress [120]. Toxic metals tend to accumulate in the fatty tissue [121], which also promote the development of AMD [59,69,122]. 

### 5.4. Other Risk Factors

The strongest demographic risk factor for the development of AMD is age because structural and functional alternations of the retina, as well as additive effects of other pathological processes, are undeniably augmented as a consequence of aging. Numerous studies showed that risk of AMD increase with age [10,115,123,124,125,126,127,128,129]. In eye response to the damages relies on the elimination of senescent cells. The production of senescent cells or the mechanisms of the removal cells lead to a detrimental hoarding of senescent cells increases with age. In elder cells many harmful changes, such as telomere erosion, damages in DNA, increase ROS production, and metabolic disturbance are observed. The long-term accumulation of senescent cells in the effect of chronic stress is a result of aging and is characteristic for age-related diseases [130].

It has been confirmed that women are more likely to suffer from AMD [118,131,132,133]. However, in cases of female sex tendency of higher progression rate to early or late AMD may be confused by such factors as differences in life expectancy or influence of sex hormones (estrogen exposure in females may even exert some protective effects as well as may be connected with favorable changes in relation to serum lipids or activity of antioxidative agents) [10]. Furthermore, the researchers indicated that the incidence of women is higher only among patients over 75 years of age. In younger groups, the incidence is the same in men and women [134]. This tendency results from the influence of hormones, especially estrogen, which most likely protects against the development of AMD [118,135].

Studies suggested that also physical activity can influence on the development of AMD. Regular exercise has positive impact on the activity of antioxidative enzymes, which may protect against the development of AMD [118,136]. Thus, physical activity generally favors antioxidative possibilities and subsequently decreases the incidence of early or late AMD. Nevertheless, this factor remains highly unreliable in the prediction of AMD progression (difficulties in precise quantification of physical activity) [10]. Even educational status has been studied; this risk factor only in a few studies was associated with the development of AMD. Education level is inversely related with the development of early [137] and late AMD [138,139]. It may be connected with the fact that higher education level usually favors better nutrition, usage of eye care services and voluntary cessation of smoking [10].

Another very important risk factor is ethnicity: in multi-ethnic studies, it has been calculated that prevalence rates of any form of AMD for different ethnic groups. In effect the lowest risk of development of AMD was in African group (2.4%) and the highest was in Caucasians (5.4%). The remaining groups have medium rates: 4.2% Hispanics and 4.6% Chinese [140]. In the African population, AMD occur less frequently than in other ethnic groups. This is probably caused by increased melanin in RPE in Africans. This higher concentration may play role of UV filters and antioxidant free radical scavengers, which may protect by the development of AMD and subsequently act as alleviating factor in the matter of AMD development and progression. Conversely, AMD appears to be more common in the Chinese population when compared to Caucasians [10,141]. The differences in the chance of progression of this disease may also result from changes in DNA in genes susceptibility for AMD [142].

Associated diseases play important role in the development of AMD. There are several diseases, which may affect the progression of this illness. Several diseases confirmed the influence in AMD progression. In a few studies it has been observed that patients after cataract surgery have higher risk of late AMD development [143,144,145]. Research suggests that during and after cataract surgery, the process of inflammation is induced and after this, medical procedure the eye is more exposed on UV light [146]. Other hypothesis points to the same risk factors determining cataract and AMD [147]. The possible influence of hypertension has been well described [125,148,149], but this disease is not always treat as risk factor of AMD [150,151,152]. Hypertension may be a modulating factor because high blood pressure is connected with lower choroidal blood flow and troubled vascular homeostasis [10]. Some studies reported the possible influence of chronic kidney disease in the development of AMD, but this correlation is unclear [149,153]. In chronic kidney disease, a decrease in the estimated glomerular filtration rate may be connected with increased possibility of developing early AMD; however, only in patients older than 65 years. Additionally a certain serum biomarker for kidney function (cystatin C) appears to be associated with the development of nAMD and early AMD [10].

A different disease, hyperthyroidism, was presented in long-term population studies [154,155]. Research showed that an increase concentration of free thyroxine (FT4) in serum was associated with the development of AMD [155]. Conversely, an increased level of the thyroid-stimulating hormone (TSH) was not correlated with this disease [154]. Hyperthyroidism is probably brought about by increasing the level of FT4 and inducing oxidation stress [156]. Therefore, hyperthyroidism may be considered an accelerative factor for oxidative metabolism and basal metabolic rate (through induction of certain mitochondrial enzymes). Some studies showed that increase levels of thyroid hormones can damage RPE cells [10,157,158]. Other studies found that diabetes correlates with the development of AMD. Usually, there is no correlation between these two diseases [152,159]. It is possible that, in diabetic patients, hyperglycemia and dyslipidemia may induce oxidative stress in retinal tissue, which disbalance the homeostasis in this eye structure [160]. Thus, hyperglycemia and dyslipidemia accompanying diabetic individuals may act as a trigger factor for inflammatory responses in retinal tissue [10]. 

The correlation between Alzheimer’s disease (AD) and AMD was studied. Due to the fact that in both diseases extracellular amyloid β-peptide deposits are produced (in AMD in drusen, in AD in extracellular amyloid β-peptide) [10,161] researchers studied the correlation between these diseases. Moreover, in both diseases, several isoforms of apolipoprotein E (APOE) are engaged. In AD, isoforms APOE Ԑ2 and Ԑ4 isoforms are associated with decreased and increased risk for AD and AMD, respectively. 

The opposite directions are reported for AMD [162,163]. Despite these similarities, studies did not show associations between these diseases. Unclear associations also occur between AMD and Parkinson’s disease (PD). In studies of PD patients treated with levodopa, the risk of AMD was lower than in patients not treated [164]. Levodopa activates the GRP143 receptor, which reduces the release of VEGF and inflammatory factors from RPE cells. This observation showed that levodopa may reduce inflammatory reactions in retina and delay the progression of AMD [10,165]. Ultimately, early AMD development may be accelerated in the event of heavy alcohol consumption and mentioned hypertension. In this context antioxidant disturbances and augmented oxidative stress persistently play a major role [113]. 

## 6. The Influence of Genetic Factors on the Development of AMD

Studies shown that many genetic factors affect in small or moderate morbidity of AMD. This is because of the interaction of both genetic and environmental factors. In specific genes heredity of this illness increases to 71% [114]. In 50–60% cases, progression of AMD is determined by polymorphisms in genes encoding *CFH*, *ARMS2*, and *Il-8* [166]. The oldest studies into the genetic background of AMD was conducted in 1988, on monozygotic twins. That was first research where severe AMD in both twins was observed [167]. In 1994, prospective studies (1986–1993) were examinated monozigotic and dizigotic twins. In effect, AMD occurred among 23 pairs of monozygotic twins [168]. 

Other studies (e.g., Familial Aggregation Studies) also confirmed genetical background. Studies showed that frequency of AMD occurrence was twice as high in people who had in first-degree relatives with that illness than in people who had no one in family tree with AMD [92]. In publications from 1999, 50 pairs of monozygotic twins and their 47 spouses were examined. All participants were from Iceland and exposure to environmental factors was similar for twins and spouses. Results showed that AMD occurred more frequently among twins than among spouses [169]. Other studies published in 2001 (Beaver Dam Eye) were conducted on patients with AMD who had siblings. After 5 years, healthy siblings which had no signs of disease before were again tested. The odds ratios (Ors) for patients were 10.32 for exudative AMD compared to controls [170]. 

Further important studies were published in 2005 in the USA. They examined pairs of twins (210 monozygotic, 181 dizygotic, and 58 singletons). Studies have divided the factors induce the development of AMD into environmental and genetic ones. The team confirmed the significance and role of genetic factors in the development of AMD. It is estimated that this influence is important for severity of AMD (46–71%) [95]. These studies confirm the involvement of genetic factors in AMD. Now, many studies have been carried out in search of specific genetic factors. These studies are presented in Table 5. 

### 6.1. CFH

This gene is located in locus 1q32-q32.1 [171], and encodes two proteins: CFH and factor H-like 1 protein (FHL-1), which are part of complement system [224,225]. Studies have shown that changes in noncoding parts of this gene are stronger correlated with the development of AMD than polymorphisms occurring in coding parts [183]. Many studies have identified a numerous of polymorphisms in this gene, which may promote the development of AMD. The most significant is *rs1061170* (named *Y402H*), which was detected in 2005 [171,172,173,174]. Later studies confirmed that the presence of *C* allele in this polymorphism significantly increases the risk of progression of AMD [175]. Furthermore, it is estimated that the proportion of this polymorphism in the development of this disease is 60%, and is the most important risk factor [176]. Other studies from 2008 indicated that *Y402H* polymorphism correlates with another (*LOC387715*, *C3*) and cigarette smoking increasing the risk of AMD [177]. Studies also indicated that *CC* variant in the *Y402H* in patients with dry AMD may have a significant effect on inflammatory regulation and affect the development of AMD [178]. Allele *CC* also affects the accumulation of macrophages after death in the tissues of eye. It is possible that polymorphism *rs1061170* affects deregulation of proinflammatory cytokines [179]. 

It was suggested that the occurrence of *rs1061170* allele does not affect neovascularization in healthy individuals, because studies have shown that the occurrence of this polymorphism does not affect changes in retinal blood vessels in healthy individuals [226]. Similar unexpected results were reported in 2015, which examined how the presence of extramacular drusen and genetic load of *rs1061170* correlated with the development of AMD. The results showed that drusen strongly correlates with AMD development, but the presence of polymorphism has no effect on the formation of drusen. The authors also stated that patients with extramacular drusen, but without AMD, could be in the control group in research on AMD risk factors [227].

Studies indicated correlation between two polymorphisms in genes *CFH rs1061170* and *HTRA1 rs3793917*, and the progression of exudative AMD [180,181]. Other polymorphisms that may increase the risk of AMD are *rs1410996*, which was found in a Caucasian population [191,228], *rs1329428*, and *rs1329421*, which were indicated by examining Spanish population [229], and also *rs551397*, *rs800292*, *rs1410996*, *rs2274700*, *rs1329424*, *rs10801555*, *rs10737680*, *rs12124794*, and *rs10733086*, which were obtained in the Chinese population [209,230]. Studies by an Iranian research group have found polymorphisms of *rs800292*, *rs2274700*, *rs1061170*, and the new *rs3753395*. All of them were correlated with AMD development [231]. Another research on the Spanish population confirmed role of haplotypes *rs800292*, *rs1061170*, and *rs800292* and show new ones—*rs529825* and *rs203674* —in the development of AMD [232]. Important polymorphisms were also obtained in *rs121913059* [224,225].

In the *CFH* gene, the protection of polymorphisms against the development of AMD are also known. In 2013, *rs6677604* was detected, which ensures correct regulation of complement system. This polymorphism is linked with lower concentration of CFH in serum [182,183,184].

### 6.2. CFHR1 and CFHR3/CFHR1

There are five CFH-related proteins, all involved in complement control. Genes encoding these proteins are located in the *1q32* locus within the tandem 355 kb of genomic region [233]. A study from 2013 demonstrated positive effect of deletion of CNP147 (*rs6677604*) on the protection against the development of AMD. This polymorphism correlates with a decrease level of CFHR1 in serum. This reduces competitive inhibition of CFH binding by competitive inhibition of CFH binding to tissue surfaces. As a result, CFH inhibits C3 convertase and protects against inflammation process [183].

Correctly, this polymorphism is *delCFHR1/CFHR3* deletion (*CNP147*), and this change affects the *CFHR3* and *CFHR1* genes and its other protective polymorphism [185]. The change was also detected in 2006 by studying European and African population [186]. Later, other studies also confirmed that this polymorphism protects against the development of AMD. An enhanced complement inhibitory effect was obtained in the presence of *delCFHR1*/*CFHR3*, induced by CFH, as the effect of alternative inhibited pathway activation [185,186,187]. 

### 6.3. ARMS2 and ARMS2/HTRA1

In this gene, the most common appears to be the polymorphism of LOC387715 (rs10490924 (A69S), located in exon 1 10q26 [234]. Numerous studies in many populations have shown that this change strongly correlates with the development of late AMD [173,174,191,192,209,229]. Other studies have shown that carriers of *TT* genotype for *rs10490924* have a 10 times higher risk for progression of AMD [172], because of the intraocular complement activation. However, precise mechanism is still unknown [188]. Studies have also confirmed the correlation of polymorphisms of *ARMS2*, *CHF*, and *C3* in increasing risk of development of AMD [177]. Important results were found in 2015, which confirmed that the presence of drusen correlates with AMD development. However, the presence of polymorphism of *ARMS2 rs10490924* is not related with the occurrence of drusen [227].

Studies confirmed that single nucleotide polymorphisms (SNPs) in the promoter region of *HtrA1* strongly correlates with the development of AMD [189,191,235]. This polymorphism increased the expression of HtrA1, but further influence on the development of AMD is unclear [189]. Studies conducted in Chinese population showed, that polymorphisms in these genes (*rs11200638* and *rs3793917*) play role in progression of AMD [209]. Other studies on the Mexican population have shown that *rs10490924* may affect the development of AMD [236]. Research conducted on the Spanish population in 2014 presented polymorphisms of *rs10490923* in *ARMS2* and *HTRA1 rs11200638*, which correlate with the development of AMD. Interestingly, both *ARMS2/HTRA1* (*rs10490923*/*rs11200638*) polymorphisms of both alleles increase the risk of AMD progression nine times [228]. 

### 6.4. Complement Factor C3

The third complement factor (*C3*) is located in the chromosome 19. Studies from 1990 have shown that gene *C3* can occur in two variants of *C3 F* and *C3 S*. *C3 S* is most common, *C3 F* is not so popular, but occur more frequently in Caucasians [28,237,238]. The presence of *rs2230199* (*Arg80Gly*, *R102G*) polymorphism within *C3* gene correlates with the development of AMD [28,192], because of the changes in the expression level and binding ability of complement factors to the ligands [195]. Other studies have also shown the effect of polymorphisms of *R102G* [191,193,194], *P314L* [194], *rs2241394* [239], *K155Q* [224], *rs6795735* [192], and *rs1047286* [236] in the development of AMD.

### 6.5. Vascular Endothelial Growth Factor VEGFA

Vascular endothelial growth factor VEGFA has locus in *6p12*. It plays an important role in angiogenesis, vasculogenesis and lymphangiogenesis. This factor is responsible for maintaining proper retinal structure by controlling the growth and maintenance of blood vessels. Polymorphisms in this gene lead to neovascularization and development of AMD [240]. Studies have shown that polymorphisms of *rs3025039* (*+936C/T*) [196,198], and *rs833061* (*−460C>*), *rs2010963* (*−634G>C*) [241], and *rs4711751* [242], correlate with the development of AMD because of the lower concentration of VEGF in serum. Conversely, polymorphism of *rs3025020* is associated with elevated VEGF level [196,197]. It is also possible that *rs699946* promotes the progression of AMD [192], while *rs699947* polymorphism is correlated with an increased concentration of VEGF, which is a risk factor for AMD [243]. Other studies suggest, that *rs943080* polymorphism protects and decreases the expression of this gene and protects against the development of this disease [242].

### 6.6. CFB

*CFB* gene is located in region 6p21.3, codes complement factor B. Studies showed that these polymorphisms: *rs641153* [244], *rs12614* (*32W*) may have protective effects against development of AMD [166,190,200]. Polymorphisms of *rs4151667* and *rs641153* (*32Q*) have different effects, identified as protective [192]. Other studies have suggested that the presence of these polymorphisms may promote the development of AMD [230,245].

### 6.7. SKIV2L

*SKIV2L* gene encodes helicase superkiller viralicidic activity 2-like enzyme. It is located in the 6p21. It participates in RNA degradation and play a role in the control of autophagy [201]. Studies have shown that *rs429608* polymorphism appeared more frequently in the control group, which suggest that it may have a protective effect against AMD development, but this mechanism is unknown [201,202]. 

### 6.8. LIPC

*LIPC* gene is located in locus *15q21.3*, encodes hepatic triglyceride lipase, an enzyme which participates in lipid metabolism converting HDL to LDL in the liver [203]. It was confirmed that the polymorphisms of *rs10468017* [190,203,204,205,206] and *rs493258* [190,191,207] correlate with high serum HDL levels, which protect against the progression of AMD [190].

### 6.9. CETP

*CETP* is the gene encoding cholesteryl ester transfer protein, which is located in the locus of 16q21 [192]. Studies have shown the contribution of two polymorphisms, which increase the risk of development of AMD, i.e., *rs2230199* [192] and *rs3764261* [179,199,204,206,208,209,210,211]. 

### 6.10. CFI

*CFI* gene encodes complement factor I (CFI) and is located in the locus of *4q25*. It plays a role in the inactivation of C3b factor, but its action is regulated by CFH. The study conducted in 2009 covered genotyping and sequencing, demonstrating the presence of *rs10033900* and its correlation with the development of AMD [213]. Next studies demonstrated the significant contribution of *rs10033900* of *TT* polymorphism in the development of AMD [191]. Additionally, the latest meta-analysis indicated that the polymorphisms of *rs10033900 T>C* and *rs2285714 C>T* could promote the progression of AMD [214].

### 6.11. IL-8

*IL-8* gene is in the *4q12-q21* locus. Studies have shown that *IL8 -251AA* polymorphism is proinflammatory and may be a risk factor for the development of AMD [215]. Later, it was also indicated that the carriers of *IL-8 +781 C/T* of SNP have higher risk of the progression of wet AMD [216]. Other studies showed that polymorphism of *rs2227306* elevates inflammation and increase angiogenesis, which may lead to the development of AMD [166,217]. 

### 6.12. GST Polymorphisms

Glutathione S-transferase (GST) enzymes, as divided into four groups (GSTA-α, GSTM-µ, GSTT-θ, GSTP-π), are responsible for catalyzing the bonding reaction of glutathione with ROS [246]. This results in water-soluble products, that can be removed from the body. The first study of the polymorphism of this gene was from 2006. The research team analyzed DNA from patients’ blood to determine changes in genes of *GSTP1*, *GSTM1* and *GSTT1*. The results from patients were significantly different from these from controls [218]. Also in 2006, another team tested a number of genes, including *GSTM1* microsomal glutathione-S-transferase 1. The results did not show *GSTM1* association with AMD development. However, as in the previous study the group was too small to explicitly deny the participation of *GSTM1* in the development of this disease [219]. 

Further studies on a larger number of individuals have shown that *GST* polymorphisms may correlate with the development of AMD. Studies confirmed that *GSTM1-null* polymorphism correlates with both dry and wet AMD [220]. In 2012, Chinese patients were conducted on the occurrence of variant *rs1695* of *GSTP1*. The results demonstrated that this allele is correlated with the development of wet AMD [223]. In the same year, *GST* polymorphism study was conducted in Iran. The results showed that *GSTM1* and *GSTT1* correlate with the higher risk of progression of AMD. *GSTM1* polymorphism is associated with the decline in the expression of protein and is a risk factor, if the activity of GST enzyme is lower, ROS cannot be effectively neutralized and affect the development of AMD [221,222].

### 6.13. Other Polymorphisms Promoting the Development of AMD

Studies indicated polymorphisms promoting the development of AMD, i.e., *rs8135665* (*SLC16A8*), *rs6795735* (*ADAMTS9-AS2*) [192], *rs17810398* and *rs17810816* (*DAPL1*) [247], *rs13095226* (*COL8A1*) [206,242], *rs1999930* (*FRK/COL10A1*) [242], *rs3094111* (*DDR1*) [248], *rs13278062* (*TNFRSF10A*) [249], *rs5888* (*SCARB1*) [250], *rs2243250* (*IL-4 -590*) [251], and *Pro197Leu* in *GPx1* [11]. Studies confirmed that polymorphisms of *rs547154* (*C2*) [244], copy number variation of *C4A* and *C4B* [247], *rs2679798* (*MYRIP*) [201], *rs174547* (*FADS1*) [192], *rs429358* (*APOE*), and *rs9621532* (*TIMP3*) [192] can play a protective role against the development of AMD.

Studies on the mitochondrial DNA show that the JTU haplogroup cluster (haplogroups in mtDNA; and changes in groups of J, T, and U) strongly correlates with the development of AMD. The SNP defining the JTU haplogroup cluster interferes with bioenergetics within the retina and may play a role in the development of AMD [173]. Studies conducted on eye tissues in 2015 showed that mtDNA damage in mice was significantly higher in mRNAs [252]. In addition, meta-analysis has shown that the polymorphisms of *mt16111*, *mt16362*, *mt16319*, *mt1736*, and *mt12007* were characteristic of the Mexican part of American population [253].

## 7. Summary and Conclusions 

This review shows current knowledge about AMD. It focused on risk factors and current mechanisms of the development of AMD. This disease may develop as a result of various factors, focused on different polymorphisms which occur in the inhabitants of different regions and environments (also in those we examined directly) and they correlate with the place of residence and the development of AMD. These results will help with future diagnosing patients from different places of residence. 

The action of chemical elements and oxidative stress are closely linked to the development of early and late AMD. Pb, Cd, and Hg had a clear impact on the late AMD. Mn and Zn have a positive impact on this AMD phase. The early phase is influenced by Pb. AMD is age-related and causes blindness in approximately 70% of patients. Pb and Cd accumulate in retinal tissues and may cause damages with participation of oxidative stress, which has consequences for AMD. Pb and Cd through DNA damage increase oxidative stress and produce reactive oxygen species in retinal pigment epithelial cells. These cells interact with basal metals, as well as toxic heavy metals. Cd in the eyes is mainly deposited in people exposed to prolonged exposure and cigarette smokers. Cd and Pb have the ability to appear at a higher concentration in the eye tissue than in the blood. 

Currently, exposure to toxic metals is lower than in the past, but Pb and Cd deposited in the eye and can promote AMD. The occurrence of chronic oxidative stress has a big impact on the human eye, because the level of antioxidant enzymes decreases with age. The eye becomes more susceptible to stress and free radicals leading to the development of inter alia AMD. 

Retina and choroid have the ability to assimilate heavy metals. Sometimes, elements such as Al, Se, and Tl are also deposited. Al concentration correlates with the development of AMD, causes damage, and the breakage of lysosomes, migration of immune cells, and inflammation, leading to tissue damage and tumor growth. Changes in the eye also cause Hg, which affect the late pathogenesis of AMD. Mn and Zn have a protective effect on the development of AMD. 

A favorable activity of metallothioneins as low molecular weight intracellular proteins is able to bind metals and scavenging of free radicals encloses counteraction against destructive changes in the cell structures and preventing apoptosis of cells which happen as after-effects of oxidative stress. Metallothioneins stimulates the processes of cellular growth, favor sedate metabolism of cells and counteract inflammatory processes. They may also play protective role in the course of degenerative diseases that grow more intense in advanced age, like AMD. Retina itself is unceasingly subjected for various types of radiation that may contribute to uncontrolled generation of free radicals. 

Three isoforms of metallothioneins, MT-1, MT-2, MT-3, were detected in retina in photoreceptors, pigment epithelium, and neurons. Especially the expression of MT-1 and MT-2 increases is due to the intensification of oxidative stress and escalation of inflammatory processes. Metallothioneins exhibit an ability of binding and detoxification of heavy metal ions and subsequently contribute to minimization of negative effects of their toxicity in cells. Conversely, the binding of Zn or Cu may act as a regulator of homeostasis and bioavailability of these microelements of certain antioxidative significance. 

The facts about MT attest the profitable activity of metallothioneins in range of protection of structures of organism (nervous system, retina) against oxidative stress and their potential to counteraction negative after-effects of AMD, as well as to the elimination of many risk factors of this disease. The presence of different protein isoforms is an important aspect of antioxidative protection beside typical antioxidative enzymes. 

AMD development is mainly correlated with changes in two types of genes: regulating inflammation process and coding proteins. In this way, the first group contains polymorphisms within *CFH*, *C3*, *CFI*. In the second group are *ARMS2* and *HTRA1*. Polymorphisms of *CETP* are correlated with AMD development. Many synergies have been identified between changes in genes of *CFH*, *HTRA1*, *CFH*, and *CRP*, *ARMS2* and *HTRA1*. Many other polymorphisms have been identified so far in both DNA and mtDNA that could promote the development of AMD. Today some polymorphisms that prevent the progression of AMD are known, e.g., in genes of *CFH*, *CFHR1*, *CFHR3*/*CFHR1*, *ARMS2*, *C3*, *VEGF*, *CFB*, *SKIV2L*, and *LIPC*.

We examined the polymorphisms of *rs3025039* (*VEGF*), *rs2243250* (*Il-4*), and *GST* to determine how they affect the development of this disease. The role of these mutation is still in question, so we should check how often they appear in patients. This information will allow us to get to know better the mechanism of AMD development. Our research aims to detect new markers of AMD and better understand the etiology of this disease. Although we expect a larger number of patients in the population of large cities, it is certain that we will encounter the effects of environmental pollution in this population. Here, such contaminants and the different types of defense mechanisms correlated with the immune response are described. 

## Figures and Tables

**Table 1:** Phenotypic, demographic, and environmental risk factors of AMD; based on Heesterbeek et al. 2020 [10].

**Risk Factor**	**Phenotypic Risk Factors**
Drusen	Predictive potential in context of AMD
	Deposits of extracellular debris are located between Bruch’s membrane and retinal pigment epithelium (RPE). Different types of drusen and its location, volume, measured area, or total number of drusen may serve as predictive factors for AMD progression. Small drusen may be connected with a low probability (0.4%) of developing late AMD within five years in patients older than 55 years. Medium or large drusen with or without accompanying pigmentary abnormalities may inform about early or intermediate AMD stage. Individuals with medium drusen are burdened with probability between 2% and 20% of developing late AMD within five years, while large drusen increases such probability to between 13% and 47%.
	Different types of drusen and possible types of AMD
	Individuals with calcified drusen have a probability of 26% of developing geographic atrophy (GA, subtype of late AMD) within 5 years. The presence of reticular pseudo-drusen is highly prevalent in fellow-eyes of individuals with unilateral neovascular AMD. Progression rate in cases of neovascular AMD is estimated at the level of 31% in a period of two years. Finally, cuticular drusen may be connected with variants of *CFH* gene, and may also be helpful in the prediction of the development of nAMD and GA (progression probabilities of 8.7–12.5% and 25–28% in a period of 5 years).
Alternations of the retinal pigment epithelium	Pigmentary changes and hyperreflective foci in context of AMD risk
	The presence of pigmentary changes in addition to drusen is connected with dramatic increase of risk of developing late AMD (probability of 47% within 5 years in cases of large drusen and accompanying pigmentary changes). Hyperreflective foci may be connected with progression rates of both nAMD (47% of eyes with hyperreflective foci develop this form of AMD after two years) and GA (approximately 50% of eyes with hyperreflective foci develop GA within a period of 28 months).
	Types of pigment epithelial detachment and AMD
	Fibrovascular and serious types are both connected with nAMD. Drusenoid type may be engaged in the progression of different kinds of AMD, as well as being connected with the development of pigmentary alternations and calcified drusen. In cases of GA, features like lesion size, number, location (increased rates of GA growth in eyes with multifocal and extrafoveal lesions compared to unifocal and foveal lesions) and shape (lesions with irregular shape are connected with a faster growth rate compared to lesions with a circular shape) are important.
Vascular and other features	Choroid vascular structures alternations and flow anomalies in the context of AMD
	There is a possibility that choroid vascular structures deplete in eyes with early AMD, nAMD, and GA, as well as that irregular choroidal vessels may be a predictive factor for the development of nAMD and GA. Similarly, flow abnormalities in the choriocapillaris surrounding the atrophic lesions may be connected with the escalation in GA growth rate. Moreover, in cases of eyes with a quiescent choroidal neovascularization, there is considerably higher risk of becoming exudative (15–18) compared to eyes lacking a precursor quiescent choroidal neovascularization.
	Outer retinal tubulations and dynamics of AMD
	Outer retinal tubulations can be present in areas of fibrosis after the development of exudative nAMD or at the border of GA. In cases of individuals with GA, the localization of outer retinal tubulations at the border of GA lesion promote slower rate of GA growth compared to individuals without outer retinal tubulations.
**Risk factor**	**Demographic and Environmental Risk Factors**
Age	The prevalence of early AMD increases from 3.5% in individuals aged 55–59 years up to 17.6% in individuals 85 years and older. Furthermore, the prevalence rates increase from 0.1% to 9.8% respectively, as well as for late AMD.
Sex	There is a tendency of a higher progression rate to early AMD and late AMD (mainly nAMD) in cases of female sex.
Smoking	Toxic compounds contained in cigarette smoke may trigger the formation of retinal oxidative stress, vascular changes in the choroidal vessels as well as the inflammation in RPE cells. Thus, smoking is connected with a two- to four-fold increased risk for any form of AMD. Smoking may also be associated with escalation in GA growth. Quitting smoking generally mitigates the risk of developing AMD; however, risk probabilities appear to be comparable to that of non-smoking individuals after as long as 20 years cessation of smoking.
Body composition	Higher body mass index may be connected with greater probability of AMD developing. Especially obese individuals (body mass index BMI > 30) are in danger of developing late AMD compared with individuals with normal weight (BMI 20–25).
Diet	The number of vegetables and fruits in a diet is considered to be a protective factor in context of AMD progression, because these components of diet are rich in such antioxidants as carotenoids and vitamins. Similarly, fish consumption (rich in protective fatty acids) tends to mitigate the risk of AMD progression.
Physical activity	Physical activity tends to be connected with lower odds of both late and early AMD, because regular exercise is considered to be positively associated with activity of antioxidative enzymes.
Education	Higher education level generally acts as alleviating factor for early AMD and late AMD development (inverse relation between education level and development of AMD).
Sunlight exposure	Sunlight exposure escalates oxidative stress in the retina and triggers the development of AMD. This factor may be unreliable (difficulties in quantification of the total amount of sunlight exposure).
Ethnicity	There are evident differences between ethnic groups in context of prevalence rates of any form of AMD (5.4% Caucasians, 4.6% Chinese, 4.2% Hispanics, 2.4% Africans).
Comorbidity	Certain accompanying diseases may exert influence on AMD development and progression, e.g., cataract surgery may increase the incidence of AMD and intensify its progression because of augmented inflammatory processes during surgery and increased exposure to ultraviolet light afterward.

**Table 2:** Immunological, genetic, and oxidative stress driven causes of AMD; based on Shughoury et al. 2022 [11].

Causative Factor	Immune Dysregulation and the Complement System Factors
Innate immune system	Immune complex deposition is engaged in the formation and biomolecular makeup of drusen. AMD pathogenesis may be mediated by localized inflammation and microglial cell recruitment.
Complement components	Terminal fragment C5b is engaged in the uprising of a membrane attack complex (MAC) along with such complement factors as C6, C7, C8, C9. Finally, MAC is responsible for disruption of the lipid bilayer that ultimately leads to cell lysis. In this context, complement factor H (*CFH* gene) on chromosome 1 is recognized as a major susceptibility locus for AMD development. In vitro knockdown of this gene may be connected with escalated MAC deposition in choroidal endothelial cells. Common loss-of-function *CFH* variants *rs1061170* (*Y402H*) and *rs1410996* constitute important genetic risk factors of AMD. Another *CFH rs121913059* (*p.Arg1210Cys*) may confer approximately a 20-fold increment in AMD threat. *Complement Factor I* (*CFI*) gene on chromosome 4 and such variants as *p.Gly119Arg* and *p.Leu131Arg* may be implicated in a reduction of CFI concentration and activity subsequently leading to the development of AMD. The variant in the complement *C3* gene of chromosome 19, *rs2230199* (*p.Arg102Gly*), is connected with disrupted CFH binding, conferring resistance to CFH-mediated inactivation. This variant, as well as the associated polymorphism *rs1047286* (*p.Pro292Leu*), exist commonly in Caucasian populations and considerably augment the risk of AMD. Another candidate factor potentially connected with AMD may be complement component 5 because of its presence in drusen, as well as in the context of elevated serum C5a levels in course of disease. In turn, variants in the gene coding complement component 9 may be engaged in progression to more advanced stages of AMD. Especially *rs34882957* (*p.Pro167Ser*) is connected with elevated serum concentration of C9 and increased polymerization rates, which results in increased MAC formation and advances in course of AMD. Contrariwise, certain rare variants in the *complement component 2* and *complement factor B* genes on chromosome 6 may act as protective factors against the development of AMD. Variants mitigating the function of coded enzymes subsequently influence on the activity of certain complement cascades and exert this beneficial effect.
**Extracellular Matrix/Metabolism of Lipids/Angiogenesis/Oxidative Stress/Multiple Pathways**	
Extracellular Matrix Remodeling	The structure of Bruch’s membrane depends on the balance between certain matrix metalloproteinases (MMPs) and their tissue inhibitors (TIMPs) (especially MMP-1, MMP-2, MMP-3, MMP-9, TIMP-1, TIMP-2, TIMP-3). TIMP-3 excess leads to impaired extracellular matrix turnover as well as pathologic thickening of Bruch’s membrane. Decreased TIMP-3 activity may lead to escalated angiogenesis. Nine rare variants in *TIMP-3* are considered to be cumulatively connected with over 30-fold increased risk of AMD (*rs5754227*, *rs713685*, *rs743751*, *rs5749482 TIMP-3* intron variants constitute particularly important risk factors). In the matter of MMPs -gene *MMP-2* appear to be significant. There are admissions that *T* allele (*TT* and *CT* genotypes) of *rs243865* polymorphism may influences protectively against AMD, whereas homozygous *CC* genotype is possibly connected with hard drusen development in the course of disease. Variants of *MMP-9* (*rs142450006*, *rs3918241*, *rs3918241*, *rs3918242*, *rs4810482*, *rs17576*, *rs17577*) appear to be connected with threat of AMD and possible progression to the form of macular neovascularization. Also *MMP-9 CA* (13-27) microsatellite expansion variant may be related with threat of the progression to macular neovascularization. Among other genes coding for extracellular matrix components, gene variant *rs140647181*, near the *Collagen Type 8 α1* (*COL8A1*) gene is considered to be a risk factor for AMD. Presumptions about connections with AMD also apply to certain rare, protein-altering variants in the *COL8A1* gene itself.
Lipid metabolism	Lipids constitute important components of drusen making up over 40% of its volume. Pathologic accumulation of lipids results in RPE and photoreceptor loss in AMD while. Oxidation of lipoproteins participate in the progression of AMD. Therefore, variants in several genes coding for proteins engaged in lipid metabolism and cholesterol transport may be considered to be risk factors of AMD. Genes involved in functionality of high density lipoprotein (HDL) appear particularly important due to its fundamental transport and anti-inflammatory significance. The *apolipoprotein E* (*APOE*) gene is recognized as possibly connected with AMD in various contexts. E.g., allelic variant *ApoE4* may exert protective effect against the disease and confers up to 40% reduction in the threat of AMD developing. Oppositely, *ApoE2* is connected with a slightly escalated risk of AMD or pathogenesis of macular neovascularization. Important modulator is hepatic lipase (LIPC) involved in intra-retinal lipid transport and regulation of plasma HDL levels. Effects are diversified from protective (against AMD development) connected with such *LIPC* variants as *rs493258*, *rs10468017*, *rs9621532* or *rs11755724* to escalating the risk of disease associated with such *LIPC* variants as *rs13095226* and *rs3748391*. Moreover, cholesteryl ester transfer protein (CETP) may be engaged in AMD due to its role in the transport of cholesterol from peripheral tissue to the liver through transfer of cholesterol esters from low-density lipoproteins to HDLs. Mainly *CETP rs3764261* is connected with escalated risk of AMD, while two intronic variants of *CETP* (*rs17231506* and *rs5817082*) are associated respectively with slightly increased and reduced risk of AMD. Another factor is ATP-binding cassette transporter A1 (ABCA1) protein because its function in the elimination of excessive tissue cholesterol by triggering the formation of HDL. The *rs1883025 ABCA1* protein seems to exert the opposite influence due to its two alleles (*C* allele connected with increment of plasma HDL levels and escalated threat of AMD and *T* allele related to decreased HDL and lower risk of AMD).
Angiogenesis	Angiogenic processes may be engaged in the development of macular neovascularization. Vascular endothelial growth factor (VEGF) is considered to be a driving force of neovascularization. Among its isoforms elevated levels of VEGF-A are connected with ocular neovascular diseases. The *T* allele of *VEGF-A rs3025000* variant augments possibility of clinical response to anti-VEGF therapy in macular neovascularization. The *VEGF-A rs3025033* variant and haplotype of *rs1570360A-rs699947A-rs3025033G-rs2146323A* may be connected with decreased threat of neovascular AMD. Finally, fibulin 5 (FBLN5) dysfunction may pose important factor in AMD pathogenesis because this extracellular matrix protein is engaged in modulation of angiogenesis and antagonizing VEGF.
Oxidative stress	Certain genetic mutations heighten susceptibility to oxidative damages that leads to photoreceptor dysfunction and AMD pathogenesis. Rare mutations in *RAD51B* gene are connected with an augmented threat of disease and individuals with AMD may characterize themselves with abnormally decreased serum concentrations of RAD51B. Additionally, the tumor necrosis factor receptor superfamily member *10A* (*TNFRSF10A*) gene mutations may increase the risk of AMD, mainly in Asian populations. Animal models confirmed that decreased expression of oxidative stress is connected with reduced RPE cell viability and escalated apoptosis. Mutations in excision *repair cross complex 6* (*ERCC6*) gene may exert certain influence on the augmentation of AMD risk because individuals with AMD may demonstrate decreased retinal ERCC6 expression. Subsequently, in such patients ERCC6 functionality in context of transcription-coupled excision repair of DNA mutations undergoes perturbation.
Genes implicated in multiple pathways	The region of chromosome 10q26 spanning high-temperature requirement factor A serine peptidase 1 (*HTRA1*) gene promoter and the age-related maculopathy susceptibility 2 (*ARMS2*) gene coding region constitutes loci of particular significance in context of AMD susceptibility. In cases of Caucasians and East Asians, *ARMS2*-*HTRA1* region together with *CFH*, is responsible for over half of the genetic threat related to AMD. *ARMS2*-*HTRA1* variants are connected with more rapid course of AMD. Certain local *ARMS2* dysfunction may be associated with oxidative damages of the retina. In turn, *HTRA1* shows implication in extracellular matrix remodeling, TGF-β cytokine signaling as well as acts as regulator of local subretinal inflammatory processes and angiogenesis. Both gene variants *HTRA1 rs11200638* and *ARMS2 rs10490924* are connected with an escalated AMD threat and younger onset of the disease. *ARMS2 rs10490924* may implicate inflammatory response due to correlation with elevated C-reactive protein levels, while G allele is connected with augmented threat of AMD progression to advanced stages. However, this variant pose as more significant risk factor of the disease in Asian populations compared to Europeans (risk allele frequencies of 40% versus 20%). This fact also gains importance in the context of a slight connection between *ARMS2* dysfunction and progression to neovascular disease. Other *ARMS*-*HTRA1* variant *rs2284665* seems to be associated with development of macular neovascularization. Finally, *HTRA1* variants *rs1049331*, *rs2293870*, and *rs2284665* are reported as connected with AMD progression, possibly through disrupted HTRA1-mediated inhibition of cellular apoptosis and insulin-like growth factor 1.

**Table 3:** Impact of toxic metals (As, Cr, Ni, Cd, Pb, Hg) on human health.

Chemical Element	Harmful Compounds	Mechanism of Action/Health Consequences of Exposition
Arsenic [As]	As_2_S_3_, AsS, AsO_6_, FeAsS	Absorption
		The most common arsenic compounds are mainly absorbed by respiratory tract and accumulated in keratin-rich structures (hair, nails, skin), and in the placenta [48].
		Health effect and biochemical impact
		Arsenic and its compounds are carcinogenic for respiratory system, skin and other organs triggering tumors, and impaired metabolic processes of hepatocytes and kidneys. Arsenic blocks sulfhydryl groups of proteins and inhibits enzymes. It can react directly with thiols, especially glutathione, cysteine, and dithiides [48].
Chromium [Cr]	Valuable III active compounds	Consequences of reactivity
		Compounds react with substances that allow the diffusion of ions (pyrophosphates, methionine, glycine, leucine, lysine, proline). They form stable protein complexes and generate the mechanisms of harmful influence of chromium [49].
		Health effect
		Cr compounds damage the digestive system, cause skin lesions, mutagenic, embryotoxic, and teratogenic effects [49].
Nickel [Ni]	Ni^2+^ compounds and ions	Physiological significance
		Nickel in low concentrations is needed for normal functioning, whereas deficiency causes a reduction of oxygen consumption by the liver and an increase of fat accumulation. Physiologically, Ni activates certain enzymes, increases the activity of humoral response, stabilizes the nucleic acids, and plays a role in lipid metabolism [50].
		Absorption and health effect
		Nickel is a major causative agent of contact allergy [50]. A total of 75–80% of inhaled nickel remains in the lungs, when administered orally 90% is excreted. Nickel allergy is most often manifested as allergic contact dermatitis (Ni-ACD). Hand eczema is also observed, resulting from prolonged exposure to Ni contained in detergents or nickel-plated items. There are known cases of vesicular eczema following the consumption of foods containing Ni [50].
Cadmium [Cd]	CdO, CdCl_2_, CdSO_4_	Absorption
		When absorbed from food, cadmium effects undergo modulation in the range of digestive tract thanks to protein agents, Zn, Cu, Ca, and Fe contained in the diet. Their low content in the food cause increases in the absorption of Cd from the gastrointestinal tract and influence its accumulation in the body [51]. The greatest amount of Cd is absorbed in the duodenum. During absorption in the enterocytes, the transport of Cd occurs (non-specific divalent metal transporter (DMT1) bivalent conveyor and metal tolerance protein (MTP1) conveyor). Absorption can also occur through calcium channels of transporters responsible for Zn transport or can be absorbed from gastrointestinal tract in the combination with cysteine or glutathione-thiol groups [52].
		Accumulation and health effect
		Cd accumulates in the liver and kidneys, which are the target organ of toxicity. In the liver, Cd induces the synthesis of low molecular weight metallothionein (MT) proteins that bind Cd (II) ions to Cd-MT complexes. In the kidneys, Cd-MT complexes are easily filtered in glomeruli and resorbed in proximal tubules, where they degrade Cd ions. These structures are particularly exposed to metal toxicity and causing subjected to damages them and resulting that may result in resorption disorders [20,21,22,43,53,54].
		AMD risk assessment
		The results suggest that people with higher levels of Cd exposure are at increased risk for AMD. Concentration of Cd in the blood is a good reflection of recent exposure, especially in exposed workers. Additionally, nicotine is one of the main risk factors for AMD development and the main source of human exposure to Cd [53].
Lead [Pb]	All Pb compounds and metallic Pb	Absorption and health effect
		Lead is mainly absorbed through respiratory and digestive tract [43,49,55]. In children exposed to Pb in the pre- or postnatal period, a number of deviations in mental development were demonstrated. The most commonly observed aggression seizures, emotional lability, memory disorders, and learning and reading difficulties [43,55]. The most common disease is saturnism. It is chronic poisoning with lead and its salts. It occurs in employees of printers, battery factories and lead paint factories [43,49]. Pb can cause high blood pressure. Through nephrotoxic activity lead may cause renal dysfunction and develop the renal hypertension. Pb can generate oxidative stress, which in turn results in dysfunction of sympathetic system. It also reduces baroreceptor sensitivity and vagus nerve tension. Pb significantly influences the conduction of stimuli in the parasympathetic system. This can result in ventricular arrhythmias or myocardial infarction [21,43,55]. Pb and Cd induce changes in vascular endothelium and thus contribute the development of atherosclerotic lesions. Lead affects the course of early and late inflammatory reactions, T and B lymphocytes, macrophages and cytotoxic cells. Bone marrow damage is a common complication of Pb poisoning. Hematopoietic disorders manifest as thrombocytopaenia, leukopenia or anemia. In advanced bone marrow failure, all marrow cell lines undergo perturbation. The higher levels of immunoglobulins, especially IgA, IgE and IgG, were also observed in patients with higher Pb. Neurological disorders after long exposure to Pb usually take the form of Pb neuropathy. It usually involves the motor neurons of the upper limbs, and in particular the radial nerves. Exposure of men to Pb results in an increased risk of spontaneous abortion in their partners. In men, gonadal dysfunction occurs in the form of decreased sperm quality [43,55].
		AMD risk assessment
		The present levels of Pb exposure in adult Americans are lower than in the past, and recent levels of exposure determined by blood lead measurements are not high enough to increase the risk of AMD. Such a hypothesis is probable, however, Pb accumulates in RPE may participate in ocular cancer as well as promote chronic disease by increasing chronic oxidative stress. As the level of antioxidant enzymes decreases with age, the retina is susceptible to stress and free radicals, which can lead to AMD development [56,57].
Mercury [Hg]	Inorganic compounds HgCl_2_, HgCn_2_, HgS	Absorption and consequence of exposure
		In people exposed to professional exposure to mercury, it penetrates through respiratory tract. A total of 80% is retained in the body. Sulfhydryl ligands (RSH) form complexes with Hg (reduced glutathione) and amino acids with cysteine and glycine. Directly high mercury poisoning occurs in cases of metallic form, from which 70% is removed to the outside of lungs. It partly penetrates into the brain [43,49].
		Accumulation and mechanism of toxicity
		Mercury remains in the blood and penetrates the blood–brain barrier and placental barrier. Consequently, it accumulates in the brain and fetal tissues. Hg^2+^ ions have the ability to form complexes with proteins and with compounds containing SH-groups. Fetal hemoglobin has a high affinity for Hg, and fetuses have a higher concentration of Hg than mothers. Due to the fact that mercury easily diffuses into lipids it is also present in breast milk of mothers. It is deposited in the hair, its concentration there is proportional to the concentration in the blood. The first mercury-invaded cell element is the cell membrane. This is due to the presence of SH-groups. Most proteins and enzymes in their composition contain amino acids of these groups, therefore Hg poisoning can interfere with enzymatic reactions [43,49]. Irrespective of the form of Hg, it is deposited in the form of a complex with metallothioneins (MT), because of the induction of MT production. Synthesis of these proteins play a key role in the detoxification process [20,21,22,43,49,54].

**Table 4:** Location of metallothioneines in the human body [88,89].

Isoform	Location of Expression
MT-1	brain (mainly astrocytes), liver, kidney, eye
MT-2	brain (mainly astrocytes), liver, kidney, eye
MT-3	brain (astrocytes, neurons, cortex, hippocampus), heart, kidney, eye
MT-4	squamous epithelia (upper gastrointestinal tract and skin), maternal deciduum

**Table 5:** Most significant polymorphisms engaged in the progression of AMD.

Gene	Polymorphism	Mechanism	Role in the AMD	Reference
*CFH*	*rs1061170 (Y402H)*	Deregulation of proinflammatory cytokines, changes in alternative complement pathway	Progression of AMD	[166,171,172,173,174,175,176,177,178,179,180,181]
	rs6677604	Regulation of complement system	Protection against AMD	[182,183,184]
*CFHR1*	*rs6677604*	Decrease level of CFHR1 in serum	Protection against AMD	[183]
*CFHR3/CFHR1*	*delCFHR1/CFHR3 (CNP147)*	Enhanced complement inhibitory activity	Protection against AMD	[185,186,187]
*ARMS2*	*rs10490924*	Activation of intraocular complement	Progression of AMD	[166,188]
*HTRA1*	*rs11200638*	Increased the expression of HtrA1	Progression of AMD	[189,190]
*C3*	*rs2230199* *(R102G)*	Changes in the expression level and binding affinity of complement factors	Progression of AMD	[28,191,192,193,194,195]
*VEGFA*	*rs3025039*	Lower concentration of VEGFA	Progression of AMD	[196,197,198]
	*rs3025020*	Elevated level of VEGFA	Progression of AMD	[196]
*CFB*	*rs4151667*, *rs641153*	Suppressing the inflammation process	Protection against AMD	[166,190,199,200]
*SKIV2L*	*rs429608*	Unknown	Protection against AMD	[201,202]
*LIPC*	*rs493258*, *rs10468017*	Elevated HDL levels	Protection against AMD	[190,191,203,204,205,206,207]
*CETP*	*rs2230199*, *rs3764261*	Changes in the concentration of cholesteryl esters in HDL	Progression of AMD	[44,179,199,204,206,208,209,210,211,212]
*CFI*	*rs10033900*	Changes in CFI production	Progression of AMD	[213,214]
*Il-8*	*IL8 −251AA*, *IL-8 +781C/T*, *rs2227306*	Increase in inflammation and angiogenesis	Progression of AMD	[166,215,216,217]
*GSTM1*	*rs1183423000*	Weaker antioxidant protection		[218,219,220,221,222]
*GSTT1*	*rs1601993659*	Weaker antioxidant protection		[218,222]
*GSTP1*	*rs1695*, *rs1138272*	Weaker antioxidant protection		[218,222,223]
